# Reasons for Unmet Need for Child and Family Health Services among Children with Special Health Care Needs with and without Medical Homes

**DOI:** 10.1371/journal.pone.0082570

**Published:** 2013-12-10

**Authors:** Jane E. Miller, Colleen N. Nugent, Dorothy Gaboda, Louise B. Russell

**Affiliations:** 1 Institute for Health, Health Care Policy, and Aging Research, Rutgers University, New Brunswick, New Jersey, United States of America; 2 Bloustein School of Planning and Public Policy, Rutgers University, New Brunswick, New Jersey, United States of America; 3 Center for State Health Policy, Rutgers University, New Brunswick, New Jersey, United States of America; 4 Department of Economics, Rutgers University, New Brunswick, New Jersey, United States of America; University of Alabama at Birmingham, United States of America

## Abstract

**Objectives:**

Medical homes, an important component of U.S. health reform, were first developed to help families of children with special health care needs (CSHCN) find and coordinate services, and reduce their children’s unmet need for health services. We hypothesize that CSHCN lacking medical homes are more likely than those with medical homes to report health system delivery or coverage problems as the specific reasons for unmet need.

**Methods:**

Data are from the 2005-2006 National Survey of Children with Special Health Care Needs (NS-CSHCN), a national, population-based survey of 40,723 CSHCN. We studied whether lacking a medical home was associated with 9 specific reasons for unmet need for 11 types of medical services, controlling for health insurance, child’s health, and sociodemographic characteristics.

**Results:**

Weighted to the national population, 17% of CSHCN reported at least one unmet health service need in the previous year. CSHCN without medical homes were 2 to 3 times as likely to report unmet need for child or family health services, and more likely to report no referral (OR= 3.3), dissatisfaction with provider (OR=2.5), service not available in area (OR= 2.1), can’t find provider who accepts insurance (OR=1.8), and health plan problems (OR=1.4) as reasons for unmet need (all p<0.05).

**Conclusions:**

CSHCN without medical homes were more likely than those with medical homes to report health system delivery or coverage reasons for unmet child health service needs. Attributable risk estimates suggest that if the 50% of CSHCN who lacked medical homes had one, overall unmet need for child health services could be reduced by as much as 35% and unmet need for family health services by 40%.

## Introduction

In 2007 the major primary care professional organizations in the U.S. proposed the patient-centered medical home as an important component of health reform, a proposal which was quickly endorsed by health plans, employers, and medical specialty groups [1–3]. Medical homes comprise a set of services that are expected to improve quality of care and reduce costs by coordinating patients’ care effectively and efficiently. They are seen as particularly important for vulnerable populations with complex medical needs, including children in low-income, immigrant, and undocumented families [4], children identified through developmental screening as needing early intervention services [5], and adults with chronic conditions, especially those who need help with activities of daily living [6].

 Medical homes were first developed for children with special health care needs (CSHCN) [7,8], who have chronic physical, developmental, behavioral, or emotional conditions that require medical care beyond that required by children generally [9]. Their greater need for services means that CSHCN have substantially higher medical expenses than other children [10,11] putting their families at increased risk for expenses that exceed 10% or even 20% of family income [12]. Although CSHCN are more likely to be insured than other children [13], they are also more likely to be underinsured [14] and to have unmet needs [15]. The burden on families is greater when the child has more special health care needs [16], their medical needs change frequently, or daily activities are limited [17]. Their care requires numerous visits to primary care doctors, specialists, and emergency rooms, and a wide array of specialized and ancillary services [16]. Such complex needs place heavy responsibility for arranging and coordinating services on families [unpublished data], who are often not well equipped for the job [18].

The Maternal and Child Health Bureau describes the functions of a medical home that serves CSHCN as to ensure that the child has a usual source of care, has a personal doctor or nurse, obtains needed referrals, receives coordinated care, and receives family-centered care [19]. Medical homes are associated with less unmet need among CSHCN, among children with disabilities [20-24] , and among U.S. children generally [25]. Lacking such help, the families of CSHCN without medical homes may have problems identifying health care providers who meet their child's needs and coordinating services among multiple providers, contributing to their unmet needs for care. 

The National Survey of Children with Special Health Care Needs (NS-CSHCN) was designed to monitor whether U.S. states are meeting targets under Title V of the Social Security Act, which helps fund coordinated systems of care for CSHCN [26-28]. In this paper we take advantage of detailed questions asked by the 2005-2006 NS-CSHCN about 9 specific reasons for unmet need for 11 medical services to explore the association between medical homes and health system delivery and coverage problems faced by CSHCN and their families. Our analysis moves beyond previous studies of the NS-CSHCN to examine the underlying reasons, reported by parents, for unmet needs for child and family health services. Specifically, we examine whether, among those with unmet need, the presence or absence of medical homes is associated with particular reasons for unmet need, in order to identify ways medical homes have served CSHCN and may be able to serve other populations with complex needs.

## Methods

The 2005-2006 NS-CSHCN is a national, population-based survey of 40,723 CSHCN conducted as part of the U.S. State and Local Areas Integrated Telephone Survey [26,29,30]. The response rate was 56%. To be included in the sample, a household had to have at least one child with special health care needs (SHCN), as defined by the CSHCN Screener developed by the Child and Adolescent Health Measurement Initiative (CAHMI). Respondents – parents or guardians who were the most knowledgeable in the household about their children’s health and care – were asked whether their children: used prescription medications; had elevated service use; had functional limitations; needed special therapies; had emotional, developmental or behavioral problems [31]. Children who met one (or more) of these criteria because of a medical, behavioral, or health condition that had lasted, or was expected to last, at least 12 months were classified as children with special health care needs. Each child in the NS-CSHCN sample had at least one SHCN; 45% had more than one.

Within households with CSHCN, one such child was randomly selected as the subject of the interview. A parent or guardian was asked about the child’s need for 15 types of medical care (Table 1); we analyzed the 11 services for which the survey asked detailed reasons for unmet needs [26]. We grouped respite care, family mental health, and genetic counseling as *family care*, the remaining 8 services as *care of the child*. We analyzed 39,582 children (97%) whose parent/guardian reported they needed at least one of these services within the last 12 months. As explained in the Text S1, our estimates of prevalence of unmet need differ from the estimates for the National Chartbook Indicator regarding the prevalence of services needed but not received, because our estimates examine 11 services whereas the Chartbook’s estimates are based on all 15 services [32,33].

**Table 1:** Percentage of cases with unmet healthcare needs and % with specified reasons for unmet need, by type of service,^*a*^ 2005-2006 National Survey of Children with Special Health Care Needs (CSHCN).

		**Types of service**														
		**Care of the child**												**Family Care**		
		Routine prevent care		Speclst care		Prevent dental		Other dental		Rx meds	Mental health	Subs abuse trtmnt**^*b*^**	Phys/occ / speech therapy	Family mental health	Genetic counsel	Respite care
# cases w/ need for service**^*c*^**		31,136		20,955		33,447		10,229		34,839	10,027	755	8,939	4,878	2,159	1,763
# cases with unmet need		710		1,096		2,332		969		578	1,458	161	1,118	946	463	804
% of *all* CSHCN who had any need***^a^***,***^d^***		1.9		2.8		6.3		2.6		1.6	3.7	0.4	3.1	2.4	1.4	2.2
% of CSHCN who *needed this service* and had unmet need^***a***,***e***^		2.4		5.4		7.7		10.6		1.8	14.8	20.7	13.5	19.3	23.8	47.8
**Reasons for unmet need*^f^*,*^g^***		**% of those with unmet need *for the specified service*, who reported the listed reason** ^***a***,***h***^														
Coverage																
Cost too much		29.8		21.4		29.3		42.1		47.7	21.6	12.8	15.1	34.1	17.2	25.7
Health plan problem		15.6		17.6		14.6		14.0		37.1	13.0	5.6	21.7	12.6	10.9	8.2
Can’t find provider who accepts insurance		3.2		7.1		9.0		8.2		2.7	7.0	7.2	3.5	5.1	3.8	6.0
Health system delivery problems																
Not convenient times/ couldn’t get appointment	11.5		12.3		10.1		8.9		1.9		7.5	5.8	6.3	10.9	7.2	5.0
Dissatisfaction w/ provider	7.3		8.3		3.8		1.9		4.4		9.7	8.5	6.9	5.3	7.6	4.0
Not available in area/ transportation problem	5.6		11.9		6.2		7.3		1.5		10.6	14.7	12.3	7.4	10.3	29.7
Doctor didn’t know how to treat or provide care	3.2		10.2		3.1		2.1		5.2		5.3	2.3	2.1	4.2	8.1	5.2
Didn’t know where to go for treatment	3.2		6.6		4.3		3.6		1.7		7.2	7.6	3.2	7.8	12.8	11.5
No referral	0.9		3.5		0.6		1.6		2.5		1.6	0.5	1.8	1.4	1.5	2.5
No insurance**^*b*^**	30.6		15.7		22.0		21.8		23.3		12.3	6.9	6.7	16.4	11.1	6.1

^a^ Weighted to population level with weights provided by Blumberg et al. 2008 [26].

^b^ Substance abuse question was not asked of 11,732 children aged 7 years and younger with valid data on covariates, so they are excluded from this column.

^c^ Unweighted N.

^d^ Denominator is all CSHCN with at least one reported service need of any kind; N= 39,582.

^e^ Percentage of respondents with unmet need for that service. Denominator is those who stated a need *for that service*.

^f^ Reasons that were not about coverage or health system delivery factors, or that pertained to only certain services, are included in the percentage of cases with unmet need (row 2) but are not broken out in detail. These include neglected/forgot appointment; treatment is ongoing; child refused to go; lack of resources at school; sick/other health issues more important; chooses not to vaccinate; didn't need/child in good health; need dentist for CSHCN; too busy/thought not important; child too young; child not at school to receive; school refused to provide; did not ask for it; vaccine shortage; and “other”

^g^ Denied coverage, didn’t know about it, and program underfunded or difficult to obtain were offered as reasons only for respite care; they are included in prevalence of unmet need but not shown separately.

^h^ Each respondent could report more than one reason for unmet need for each type of service.

^i^ Excluded from “coverage” category of reasons because it is too closely related to the independent variables used to measure service delivery. See text for more detail.

Children who had not received some or all of a needed service were classified as having unmet need for that service (Figure S1). The survey asked why care had not been received; a list of reasons was available as prompts (see Table 1 for wording) and respondents could report more than one reason for each type of care. In the tables and descriptions below, we show 9 specific reasons that relate to health system delivery and insurance problems. "Health plan problems" were not defined on the survey, but comprised a category that interviewers used when respondents’ answers suggested that insurance was a problem. For example, responses such as "insurance does not cover that service," "the maximum benefits for that type of service were reached," "I did not know whether or how much of the costs would be covered," or "insurance does not cover enough of the cost" could have been coded as health plan problems [34]. Exploratory factor analysis (not shown) showed that for most types of child or family health services, "health plan problems" loaded with coverage issues (costs too much, no insurance, can't find provider who accepts insurance). “No insurance” was not included in the analysis of reasons for unmet need because by definition it is closely related to insurance status and insurance gap, two of our covariates (see below).

We used the medical home measure defined by the Maternal and Child Health Bureau’s “Core Outcome #2,” which is calculated from survey questions about whether the child has a usual source of care, has a personal doctor or nurse, is able to obtain needed referrals, receives coordinated care, and receives family-centered care [19]. Children whose parents reported that they did not need referrals, care coordination, or family-centered care were classified as having met those components. Children for whom all five subcomponents were reported were classified as having a medical home; those with four or fewer components were counted as not having a medical home. Cases that were missing on one or more of the components were classified as missing medical home information, which was included as a separate category in the regressions.

We controlled for 3 measures of the child’s health status, all based on parental report. First is the classification developed by Bramlett and colleagues [16], which differentiates subgroups of CSHCN based on health status and complexity of medical need, using only information on the five SHCN available from the CAHMI CSHCN Screener. The second measure follows the NS-CSHCN National Chartbook’s calculation of the child’s functional ability [32]. It combines questionnaire items regarding "how often" and “how much” the child’s conditions affected daily activities: consistently, often a great deal; moderately, some of the time; and never. The third uses a questionnaire item capturing the stability of the child’s health care needs, which could change “all the time”, change “once in a while,” or be stable. 

We also controlled for two aspects of health insurance: type of insurance at the time of interview (public, private or other comprehensive insurance, both public and private, or no insurance); and an indicator of whether those covered at the time of interview had experienced gaps in health insurance in the preceding 12 months – the period covered by the measures of unmet medical needs.

In addition, we controlled for the following demographic and socioeconomic covariates: family income as a percentage of the Federal Poverty Level, highest educational attainment of any adult in the household, family structure, number of children in the household (overall and with SHCN), primary language spoken at home, urban/rural residence, race/ethnicity, gender, and age of the child, coded as shown in Table 2. These variables were included in the models because they could potentially confound the association between medical home and reasons for unmet need for child or family health services. For example, lack of health insurance, low income, low parental education, racial/ethnic minority status, and a primary language other than English are each associated with lower than average rates of having a medical home [20] and with reporting unmet needs for health services among CSHCN [10].

**Table 2:** Health system delivery, health status, and sociodemographic characteristics of analytic sample, *^a^* 2005-2006 National Survey of Children with Special Health Care Needs (CSHCN).

		CSHCN w/ need for 1+ child services**^*b*^**		CSHCN w/ need for 1+ family services		
	Unweighted N Analytic sample (39,582)	% of children w/ no unmet needs**^*c*^** (N=33,967)	% of children w/ 1+ unmet needs **^*c*^** (N=5,575)	% of children w/ no unmet needs **^*c*^** (N=5,042)	% of children w/ 1+ unmet needs **^*c*^** (N=1,864)	
All CSHCN		85.2	14.8	72.3	27.7	
**HEALTH SYSTEM**						
Medical home		**p<.01^*d*^**		**p<.01 ^*d*^**		
No	19,329	46.7	70.6	65.2	79.3	
Yes	18,591	49.2	22.7	31.7	11.9	
Missing	1,662	4.1	6.8	3.2	8.8	
Type of health insurance		**p<.01 ^*d*^**		**p<.01 ^*d*^**		
Private/other comprehensive	25,956	64.8	43.5	48.3	33.9	
Public	9,406	26.0	37.8	37.5	44.5	
Both private & public	2,857	7.1	8.6	11.1	13.7	
Uninsured	1,363	2.2	10.2	3.1	7.9	
Continuity of insurance in preceding year		**p<.01 ^*d*^**		**p<.01 ^*d*^**		
Insured but not continuously	37,678	95.9	87.9	94.2	88.4	
Continuously insured/uninsured	1,904	4.1	12.1	5.8	11.6	
**HEALTH STATUS**						
Type of SHCN**^*e*^**		**p<.01 ^*d*^**		**p<.01 ^*d*^**		
Functional limitations, alone or w/ other SHCN	8,440	18.7	36.5	37.8	52.6	
Prescription medication & elevated service use	8,491	21.3	19.1	32.9	20.2	
Elevated service use only	5,353	12.4	21.6	18.4	22.3	
Prescription med only	17,298	47.6	22.8	11.0	4.9	
Child’s daily activities are		**p<.01 ^*d*^**		**p<.01 ^*d*^**		
Consistently affected, often a great deal	8,912	20.3	43.0	45.1		66.2
Moderately affected, some of the time	15,278	38.7	38.4	39.3		25.9
Never affected	15,392	41.0	18.6	15.6		7.9
Child’s health care needs		**p<.01 ^*d*^**		**p<.01^*d*^**		
Change all the time	2,190	5.2	11.6	13.5	19.7	
Change only once in a while	10,632	26.3	36.2	36.1	38.0	
Are usually stable	26,691	68.3	51.9	50.2	42.0	
None of the above	69	0.1	0.3	0.2	0.3	
**SOCIODEMOGRAPHIC**						
Family income (% of Federal Poverty Level; FPL) **^*f*^**		**p<.01^*d*^**		**p<.01^*d*^**		
<133% of FPL	8,765	24.1	41.4	32.1	41.6	
133% to 199% of FPL	5,528	12.7	19.8	14.2	19.0	
200-299% of FPL	7,110	16.1	15.9	15.9	14.3	
300-399% of FPL	6,096	15.1	9.2	12.1	10.0	
400%+ of FPL	12,083	32.1	13.8	25.7	15.0	
Highest educ. attainment among adults in family		**p<.01** ^d^		**p=.90^*d*^**		
<=high school graduate	8,009	28.4	36.1	31.9	32.2	
More than high school	31,573	71.6	63.9	68.1	67.8	
Language		**p<.01^*d*^**		**p.36^*d*^**		
English	38,337	95.7	94.3	95.4	94.2	
Non-English	1,245	4.3	5.7	4.6	5.8	
Family structure		**p<.01^*d*^**		**p<.01^*d*^**		
Two parent	26,078	64.9	51.2	52.9		42.6
Single mother	9,881	26.6	38.7	33.4		43.3
Other	2,070	4.9	4.9	7.5		7.5
Missing	1,553	3.5	5.3	6.2		6.6
# CSHCN in family		**p=.03^*d*^**		**p=.96^*d*^**		
1	31,508	67.8	64.9	66.5		66.4
2 or more	8,074	32.2	35.1	33.5		33.6
# non-CSHCN in family		**p<.01^*d*^**		**p=.56^*d*^**		
none	18,618	50.1	53.8	59.1		61.4
1	13,290	32.0	28.4	26.1		24.3
2 or more	7,674	18.0	17.9	14.8		14.2
Race/ethnicity		**p<.01^*d*^**		**p<.01^*d*^**		
Non-Hispanic white	28,587	67.2	58.1	65.4		57.4
Non-Hispanic black	4,034	15.5	18.4	15.5		19.8
Hispanic	3,849	10.8	15.6	12		14.2
Other	3,112	6.5	7.9	7.1		8.6
Age of index child (yrs)	.	**p<.01 ^*d*^**		**p=.20 ^*d*^**		
5 or younger	7,189	21.6	16.4	15.7		18.2
6-11	14,733	37.6	35.9	35.2		36.8
12-17	17,660	40.7	47.7	49.0		45.0
Gender of child		**p=.33^*d*^**		**p=.21 ^*d*^**		
Boy	23,509	59.1	60.3	60.3		63.1
Girl	16,073	40.9	39.7	39.7		36.9
Residence		**p=.11 ^*d*^**		**p=.69 ^*d*^**		
Rural	5,971	15.5	16.4	13.9		14.3
Urban	21,361	74.1	74.4	74.1		74.6
Missing	12,250	10.4	9.2	12.1		11.2

^a^ Sample includes only those children with reported need for or use of at least one of the health services listed in Table 1 who were not missing data on any covariate other than medical home, family structure, or residence (as the table shows, missing categories were defined for the named covariates). See methods section.

^b^ Each child could have unmet needs for child health services, family health services, or both.

^c^ Weighted to population level with weights provided by Blumberg et al. 2008 [26].

^d^ P-values are based on a chi-squared test.

^e^ Following Bramlett et als’ [16] classification of the SHCN identified by the CAHMI Screener [31].

^f^ Based on imputed income variable available in the public use data set.

### Statistical Methods

For each of the 9 reasons for unmet need, we estimated a set of multivariable logistic regressions among children who had unmet need for one (or more) of the 8 child health services and a separate set of regressions for unmet need for one (or more) of the 3 family health services. This approach yielded a total of 18 regressions. Each regression estimated the relative odds of reporting that specific reason for unmet need for that type of service if the child lacked vs. had a medical home, controlling for insurance coverage, child’s health status, and sociodemographic characteristics. 

We used the imputed income variable provided in the public use data set. To retain the substantial number of children who were missing values for medical home, family structure, or rural/urban location, we created separate “missing” categories for those covariates (Table 2). A separate category for children with missing responses for a covariate allows them to remain in the analysis and to contribute to the estimates for other covariates without biasing the results for valid responses. Children who were missing values for race/ethnicity (N=191), insurance gaps (N=160), activity limitation (N=117), stability of health condition (N=107), insurance type (N=89), educational attainment (N=82), gender (N=75), or language (N=27) were dropped from the analysis because each of those variables was missing for less than 1% of the sample. A total of 715 children (1.8% of the sample) were excluded on this basis, which is less than the sum of the component Ns because some children were missing more than one of those variables. 

To investigate the extent of multicollinearity among certain sets of predictor variables, we calculated variance inflation factors (VIFs) [35]. VIFs among the three health status measures were all less than 2, which is below the cutoff of 2.5 used to identify problematic levels of collinearity [35]. The VIFs between education and income categories were also below 2.

The NS-CSHCN used a complex sample design that drew a representative sample of at least 750 CSHCN from each of the 50 U.S. states and the District of Columbia. Except as noted, all statistics were weighted to the national population of CSHCN using the child interview weights [26] and the svy commands with subpop option in Stata 10.1 [36], which adjusted for the complex sample design. 

## Results

Overall, of 39,582 children who needed at least one of the 11 services, 17% reported unmet needs. Table 1, top row, reports the number of children who needed each service. The numbers vary because some children did not need the service or because of item non-response (less than 0.2%; see second row of Table S1 for details on non-response). 

As a fraction of those children who needed the specific service, unmet need ranged widely (4^th^ row), from 1.8% of children who needed prescription medications and 2.4% of those who needed routine preventive care to 14% for physical, occupational or speech therapy, 15% for mental health services and 21% for substance abuse treatment. Unmet need was highest for family services: 48% of those who needed respite services, 19% for family mental health services, and 24% for genetic counseling. 

Table 2 shows whether each covariate used in the regressions is associated with unmet need for child and family services. We present this information in lieu of 18 separate contrasts, one for each reason/service group since virtually all families of children with unmet needs reported at least one reason for those unmet needs. Compared with CSHCN who obtained all needed services, those with unmet needs were less likely to have a medical home or to be insured continuously, and more likely to have functional limitations, elevated service needs, health conditions that were more severe and unstable, be older, racial/ethnic minorities, and to come from lower income or single-mother families (all p<.05). 

Consistent with previous studies [20,37] we found that, compared with those who had medical homes, children who lacked a medical home had 2 to 3 times the odds of unmet need for child and family services, even when insurance, health status, and demographics were taken into account (results available from authors).

### Multivariable analysis of reasons for unmet need

**Figure 1:**
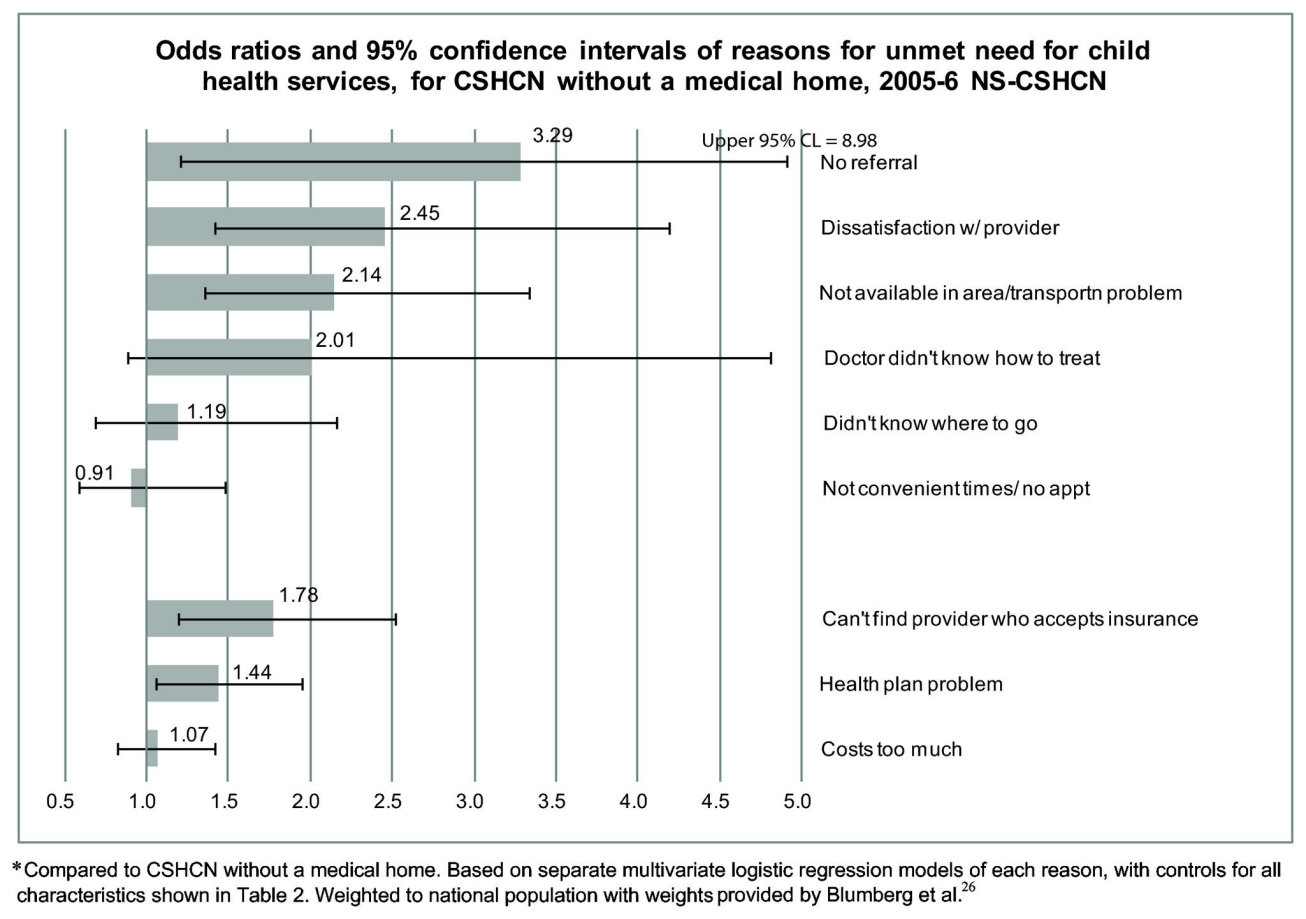
Odds ratios and 95% confidence intervals of reasons for unmet need for child health services, for CSHCN without compared to those with a medical home, 2005-6 NS-CSHCN. Compared to CSHCN with a medical home. Controlling for all variables shown in Table 2.

Figure 1 presents odds ratios (OR) from multivariable regressions of each of the 9 reasons for unmet need for *children’s medical services* for the 5,521 CSHCN who reported valid reasons for unmet need for child services. As shown in the top 6 bars, after controlling for insurance, child’s health status, and sociodemographic characteristics, lack of a medical home was associated with higher odds of unmet need for several health system delivery factors: no referral (OR =3.3; 95% CI: 1.2, 9.0), dissatisfaction with provider (OR=2.5; 95% CI: 1.4, 4.2), and problems with availability of service/transportation (OR=2.1; 95% CI: 1.4, 3.4). Of the coverage-related reasons (Figure 1, bottom 3 bars), inability to find provider who accepts their health insurance (OR=1.8; 95% CI: 1.2, 2.7) and health plan problems (OR=1.4; 95% CI: 1.1, 2.0) were associated with lack of a medical home. Detailed regression results for all models and covariates are in Table S2. 

To examine whether medical homes are associated with larger or smaller benefits for the most vulnerable, complex cases, we tested interactions between medical home and each of the three measures of child’s health status (stability, functional limitations, or severity based on the Bramlett classification). We estimated separate interaction models for each of four outcomes: indicators of (1) any of the three coverage reasons or (2) any of the six health system delivery reasons separately for (a) child or (b) family health services. None of the interactions were statistically significant in any of the models (not shown). 

Fewer families (N=1,823) reported valid reasons for unmet needs for family services. The only reason for unmet family service needs that was associated with lacking a medical home was doctor not knowing how to treat the child's condition (OR=7.5; 95% CI: 2.1, 27.6, Table S3).

## Discussion

Our multivariable analysis of data from the 2005-2006 National Survey of Children with Special Health Care Needs (NS-CSHCN) shows that several coverage and health system delivery problems were commonly reported reasons for unmet medical need among children without a medical home. Lack of a medical home was associated with higher unmet need due to lack of referrals, dissatisfaction with services, transportation problems or unavailability of the service in the respondent's area, difficulty finding a health care provider who accepts the child’s insurance, and health plan problems. These results suggest that medical homes may play a role in helping families identify suitable providers, that they provide the necessary referrals, as they are expected to do, and thus that they appear to facilitate families' efforts to negotiate the health system.

Ensuring that all CSHCN have medical homes could make a substantial difference in unmet need in this population. Attributable risk calculations based on our multivariable results show that if the 50% of CSHCN who lacked medical homes had one, overall unmet need for child health services could be reduced by as much as 35% [38]. The corresponding estimate for family health services is 40%. Among CSHCN with one or more unmet child service needs, fully 70% lacked a medical home. Attributable risk estimates for individual reasons suggest that if those families had medical homes, there could be sizeable reductions in unmet need due to lack of referral (61%), dissatisfaction (48%), problems with geographic availability of services in their area (42%), inability to find a provider who accepts their type of insurance (33%), and health plan problems (19%). These estimates provide upper bounds on the potential reductions in unmet need, and could be achieved only if the entire association between medical homes and unmet need were causal. 

In general, medical homes are more common among children with higher family incomes, with insurance [4], and with the least severe conditions [20,37]. That pattern suggests that the lower unmet need among CSHCN with medical homes could be at least partly due to selection – children with fewer needs are more likely than those with greater needs to report having a medical home – rather than to anything medical homes do. Selection of healthier patients has also been identified as a shortcoming of the medical home concept applied in Ontario [39]. We cannot rule out this possibility. We did, however, control for child’s health status and for an extensive list of other covariates in the regressions. We also tested interactions between medical home and child’s health status; none were statistically significant. These results suggest, but do not prove, that selection is not the reason for the associations we found between reasons for unmet need and lacking a medical home.

Consistent with other studies, we found that lack of insurance was an important reason for unmet need, with uninsured children 2.7 times as likely to report cost problems as those with private insurance (detailed results in Table S2) [40]. Although only 3.4% of CSHCN were uninsured at the time of interview, 8.2% were uninsured at some point during the preceding year – the period to which the unmet needs estimates pertain. CSHCN who had insurance gaps were nearly as likely as the uninsured to report cost problems as reasons for unmet needs [41]. Among those with continuous insurance, type of insurance was associated with several cost and coverage reasons. Like other studies [42-44], we found that CSHCN with public insurance were 2 to 3 times as likely as the privately-insured to report difficulty finding a provider who accepted their insurance, but only about two-thirds as likely to report high cost as a reason for unmet need. 

Medical homes and insurance appear to operate on overlapping sets of reasons for unmet need: medical homes address both health system delivery and coverage issues, while insurance addresses coverage-related problems. This pattern is consistent with the purpose of medical homes, which were created to help coordinate services for CSHCN and help their families negotiate the health care system, including locating providers who accept their insurance. Insurance is, however, related to having a medical home: only 25% of uninsured CSHCN and 37% of those with public insurance had a medical home, compared to more than half the privately insured. This suggests that those groups are susceptible to unmet need for both health system delivery and coverage reasons, and would benefit from special policy efforts to improve their access to medical homes.

Our analysis does not address how medical homes affect medical expenditures. Using the same dataset, the 2005-6 NS-CSHCN, Porterfield and DeRigne found that families’ out-of-pocket costs were lower if the CSHCN had a medical home [45]. Although their results appear to conflict with our findings that costs were not a reason for unmet need among CSHCN lacking a medical home, the two studies address different concepts: Our work concerns parents’ perceptions of why they chose *not* to obtain needed health services for their children, while Porterfield and DeRigne analyzed reported out-of-pocket expenditures on health services for those who *did* obtain those services. In the general pediatric population, Romaire et al. found that higher expenditures for prescription medications, outpatient and dental care were offset by lower expenditures for other types of care, with the result that total expenditures were the same for children with medical homes and those without [25]. 

This study has several strengths: The NS-CSHCN is a large, nationally representative sample of CSHCN, the population for which medical homes were originally developed [7,8]. The survey’s extensive detail on the extent of, and reasons for, unmet need for specific services allowed us to analyze reasons for unmet need, even for services required by only a small share of CSHCN. The NS-CSHCN also contains a thoroughly tested measure of medical home, created over several decades by experts associated with the Maternal and Child Health Bureau. Finally, several measures of child health status developed by the American Academy of Pediatrics permitted testing of whether medical homes work for CSHCN with more severe needs. 

The study also has limitations. Health status and need for services are based on parental report, which may overstate or understate their prevalence [46]. Problems getting care may be understated since the survey did not ask families that obtained all needed care whether they had difficulty obtaining that care. The measure of medical home used here is also based on parental report of received services, which may differ from the services offered by practices, as assessed against national standards such as the National Committee on Quality Assurance [47]. Since the items used to identify lack of a medical home and the reports of unmet need for child or family health services are both based on respondent perceptions of services for the CSHCN, it might be that the association between medical homes and unmet need in part reflects a general dissatisfaction with services. The number of cases reporting certain of the specific reasons for unmet need (e.g., lack of referral) is small, suggesting that the estimate of the odds ratio is imprecise, which is confirmed by the wide confidence intervals around those estimates. Finally, the NS-CSHCN included only families with landlines, excluding the 8% of children nationally who lived in households with only wireless phones in 2005-2006, and the 2% with no phone [48].

The concept of medical homes is being refined and enlarged as, spurred by U.S. health reform efforts, they move into mainstream care [49]. The experience of children with special health care needs can be instructive in this process, because these children and their families face especially difficult problems dealing with the medical system, and medical homes have been successful in substantially reducing these problems. Future research should compare patients' and providers' perceptions of medical home services, and should continue to investigate which components of medical homes are most critical for reducing unmet need associated with health system problems. Even as such future work contributes to our understanding of medical homes, however, our study adds to previous research showing that medical homes are an important way to improve medical care for children with special health care needs.

## Supporting Information

Text S1 **Supporting text for “Reasons for Unmet Need for Child and Family Health Services among Children with Special Health Care Needs with and without Medical Homes”.**
(DOC)

Figure S1 **Depicts the sequence of questions used to collect information about need for services, unmet need for services, and reasons for unmet need for services and the number of cases dropped at each level.**
(EPS)

Table S1 **Number of cases with missing values on service need, use, or reasons, by type of service.**
(DOC)

Table S2 **Estimated odds ratios of unmet need for child services for each individual reason.**
(DOC)

Table S3 **Estimated odds ratios of unmet need for family services for each individual reason.**
(DOC)
