# Supplementary material for: Reasons for Unmet Need for Child and Family Health Services among Children with Special Health Care Needs with and without Medical Homes
Source: PLoS One. 2013 Dec 10;8(12):e82570. doi: 10.1371/journal.pone.0082570 (PMC3858312; doi:10.1371/journal.pone.0082570)
Supplement: Text S1 — Supporting text for “Reasons for Unmet Need for Child and Family Health Services among Children with Special Health Care Needs with and without Medical Homes”. (DOC) [file pone.0082570.s001.doc]

Supporting Information for “Reasons for Unmet Need for Child and Family Health Services among Children with Special Health Care Needs with and without Medical Homes”

**Unmet need measure**

Our measures of unmet need for the child include only the 11 services for which the survey asked detailed reasons for unmet need.[Error: Reference source not found] Our “child services” indicator included routine preventive care; specialist care; preventive dental care; other dental care; prescription medicines; occupational therapy, physical therapy, or speech therapy; mental health care or counseling; substance abuse treatment or counseling.

Our estimates of unmet need differ from those based on the NS-CSHCN National Indicator #6, which is based on information about 15 different health care services or equipment during the past 12 months. Those services include: routine preventive care; specialist care; preventive dental care; other dental care; prescription medicines; occupational therapy, physical therapy, or speech therapy; mental health care or counseling; substance abuse treatment or counseling; home health care; vision care or eyeglasses; hearing aids or hearing care; mobility aids or devices; communication aids or devices; disposable medical supplies; durable medical equipment [[[1]](#endnote-2)]. Thus, Indicator #6 includes all child services in our measure, plus home health care and six types of equipment for which information on reasons for unmet need was not collected.

Our measure of family unmet need includes the same three services as the National Indicator #7 (Unmet Need for Family Support Services): “family services” included respite care, family mental health, and genetic counseling. Prevalence estimates may differ slightly because of differences in sample size due to the fact that the national estimates include all children as the denominator, whereas we include only those who report a need for one or more of these services, and that we exclude children with missing values on other covariates.

**Child and family services**

We aggregated 8 child services (left most columns of Table S1) and 3 family services (right column of Table S1) when determining which cases to include in the analyses. Children for whom all of the questions about services in either of those groupings were missing, don’t know, or refused were omitted from that step in the analysis. If the respondent answered “yes” or “no” to ANY of the services in that group, the child was retained in that analysis.

**Attributable risk calculations**

Attributable risk is calculated *AR* = [*p*(RR – 1)]/[(*p*[RR – 1]) + 1] × 100, [[[2]](#endnote-3)] where RR is the relative risk of the outcome (e.g., unmet need) for those without a medical home compared to those with a medical home, and *p* is the proportion of the population who lack a medical home.

The relative risk (RR) used in the attributable risk formula is calculated: RR = OR/[(1 – *p_u_*) + (OR × *p_u_*)], [[[3]](#endnote-4)] where OR is the odds ratio from the logistic regression (Tables S2 and S3), and *p_u_* is the prevalence of the outcome among CSHCN without the risk factor of interest (in our case, those WITH a medical home).

**Sample size for multivariate analysis of unmet need and reasons for unmet need**

Table S1 details the number of cases for whom information was missing/don’t know (“DK”) or refused for specific types of services named in the column headings.

Figure S1 depicts the sequence of questions used to collect information about

- Need for each of the 11 types of services listed in Table 1; top row of diagram
- Unmet need, among those with a need for each of those services; middle row of diagram
- Reasons for unmet need, among those with unmet need for each of those services; bottom row of diagram.

**References to supplemental information file**

1. Child and Adolescent Health Measurement Initiative (CAMHI) (2008) 2005/2006 National Survey of Children with Special Health Care Needs, indicator and outcome measures SPSS code and documentation, version 1.0. Data Resource Center for Child and Adolescent Health. Available <http://childhealthdata.org/docs/cshcn/0506_cshcn-spss_final_508-pdf.pdf> Accessed September 2011. [↑](#endnote-ref-2)
2. Lilienfeld DE, Stolley PD (1994) Foundations of epidemiology 3rd ed. New York: Oxford University Press. 371 p. [↑](#endnote-ref-3)
3. Zhang J, Yu KF (1998) What’s the relative risk? A method of correcting the odds ratio in cohort studies of common outcomes. JAMA 280:1690–91. [↑](#endnote-ref-4)
