# Supplementary material for: Reasons for Unmet Need for Child and Family Health Services among Children with Special Health Care Needs with and without Medical Homes
Source: PLoS One. 2013 Dec 10;8(12):e82570. doi: 10.1371/journal.pone.0082570 (PMC3858312; doi:10.1371/journal.pone.0082570)
Supplement: Table S1 — Number of cases with missing values on service need, use, or reasons, by type of service. (DOC) [file pone.0082570.s003.doc]

| Table S1. Number of cases with missing values on service need, use, or reasons, by type of service 2005-2006 National Survey of Children with Special Health Care Needs (CSHCN) | | | | | | | | | | | |
| --- | --- | --- | --- | --- | --- | --- | --- | --- | --- | --- | --- |
|  | **Types of service** | | | | | | | | | | |
|  | **Care of the child** | | | | | | | | **Family Care** | | |
|  | Routine prevntive care | Speclst care | Prevnt dental | Other dental | Rx meds | Mental health | Subs abuse trtmnt[[1]](#footnote-2) | Phys/occ/ speech therapy | Family mental health | Genetic couns | Respite care |
| Unweighted # cases w/ need for service | 31,136 | 20,955 | 33,447 | 10,229 | 34,839 | 10,027 | 755 | 8,939 | 4,878 | 2,159 | 1,763 |
| # cases DK/refused for service need | 91 | 73 | 64 | 90 | 54 | 59 | 21 | 52 | 29 | 51 | 8 |
| # cases with unmet need | 710 | 1,096 | 2,332 | 969 | 578 | 1,458 | 161 | 1,118 | 946 | 463 | 804 |
| # cases DK/refused for unmet need | 26 | 56 | 19 | 8 | 11 | 72 | 9 | 54 | 33 | 18 | 6 |
| # cases DK/refused for reasons for unmet need | 18 | 19 | 24 | 10 | 2 | 19 | 3 | 10 | 24 | 25 | 16 |

1. Substance abuse question was not asked of 11,732 children aged 7 years and younger with valid data on covariates, so they are excluded from this column. [↑](#footnote-ref-2)
