# Supplementary material for: Reasons for Unmet Need for Child and Family Health Services among Children with Special Health Care Needs with and without Medical Homes
Source: PLoS One. 2013 Dec 10;8(12):e82570. doi: 10.1371/journal.pone.0082570 (PMC3858312; doi:10.1371/journal.pone.0082570)
Supplement: Table S2 — Estimated odds ratios of unmet need for child services for each individual reason. (DOC) [file pone.0082570.s004.doc]

| Table S2. Estimated odds ratios (OR) of unmet need for child services for each individual reason , 2005-2006 National Survey of Children with Special Health Care Needs (N=5,521) | | | | | | | | | | | | | | | | | | |
| --- | --- | --- | --- | --- | --- | --- | --- | --- | --- | --- | --- | --- | --- | --- | --- | --- | --- | --- |
|  | **No referral** | | **Dissatisfaction w/ prov** | | **Doc didn't know how to treat** | | **Not avail in area / transp prob** | | **Health plan problem** | | **Didn't know where to go** | | **Not conv times / no appt** | | **Costs too much** | | **Can't find provider who accepts insurance** | |
|  | OR | p | OR | p | OR | p | OR | p | OR | p | OR | p | OR | p | OR | p | OR | p |
| No medical home | 3.29 | 0.02 | 2.45 | 0.00 | 2.01 | 0.10 | 2.14 | 0.00 | 1.44 | 0.02 | 1.19 | 0.54 | 0.91 | 0.67 | 1.07 | 0.60 | 1.78 | 0.01 |
| Medical home missing | 3.15 | 0.07 | 1.55 | 0.23 | 1.45 | 0.48 | 1.58 | 0.19 | 1.15 | 0.60 | 0.70 | 0.42 | 1.01 | 0.97 | 0.99 | 0.97 | 2.02 | 0.05 |
| Public | 2.04 | 0.04 | 0.90 | 0.69 | 1.26 | 0.36 | 1.49 | 0.06 | 0.72 | 0.05 | 1.71 | 0.02 | 0.86 | 0.41 | 0.34 | 0.00 | 2.94 | 0.00 |
| Private & public | 0.90 | 0.83 | 1.10 | 0.75 | 1.61 | 0.20 | 1.49 | 0.15 | 0.75 | 0.23 | 1.26 | 0.46 | 0.98 | 0.95 | 0.43 | 0.00 | 2.16 | 0.02 |
| Uninsured | 0.54 | 0.17 | 0.35 | 0.00 | 0.96 | 0.91 | 0.74 | 0.35 | 0.74 | 0.16 | 1.17 | 0.62 | 0.31 | 0.00 | 2.66 | 0.00 | 0.58 | 0.14 |
| Insured but with gaps | 0.65 | 0.31 | 0.81 | 0.45 | 1.26 | 0.50 | 0.63 | 0.05 | 1.36 | 0.08 | 0.80 | 0.49 | 0.63 | 0.06 | 2.15 | 0.00 | 0.76 | 0.25 |
| Func limitations, alone or w/ other SHCN | 0.82 | 0.71 | 1.46 | 0.30 | 2.47 | 0.02 | 2.15 | 0.01 | 0.97 | 0.86 | 1.00 | 0.99 | 1.54 | 0.09 | 0.83 | 0.29 | 0.96 | 0.86 |
| Prescription meds & elevated service use | 1.05 | 0.93 | 1.10 | 0.76 | 1.66 | 0.22 | 1.51 | 0.15 | 0.85 | 0.37 | 1.32 | 0.41 | 1.41 | 0.14 | 0.87 | 0.38 | 0.79 | 0.35 |
| Elevated service use only | 0.77 | 0.62 | 2.09 | 0.05 | 2.24 | 0.02 | 1.26 | 0.41 | 0.78 | 0.25 | 1.01 | 0.98 | 1.18 | 0.47 | 0.80 | 0.22 | 0.88 | 0.63 |
| Consistently affected, often a great deal | 1.45 | 0.53 | 1.46 | 0.25 | 1.55 | 0.21 | 1.15 | 0.60 | 0.90 | 0.60 | 2.07 | 0.02 | 1.02 | 0.93 | 1.28 | 0.17 | 1.09 | 0.73 |
| Moderately affected, some of the time | 1.39 | 0.57 | 2.01 | 0.03 | 1.24 | 0.52 | 0.81 | 0.41 | 0.88 | 0.51 | 1.25 | 0.47 | 1.00 | 1.00 | 1.18 | 0.30 | 0.77 | 0.26 |
| Change all the time | 2.17 | 0.09 | 1.03 | 0.91 | 1.11 | 0.71 | 1.37 | 0.13 | 0.87 | 0.45 | 0.71 | 0.21 | 0.83 | 0.44 | 0.77 | 0.12 | 0.59 | 0.05 |
| Change only once in a while | 0.64 | 0.18 | 1.23 | 0.31 | 1.32 | 0.19 | 1.55 | 0.01 | 1.41 | 0.01 | 1.06 | 0.76 | 0.81 | 0.19 | 0.93 | 0.54 | 0.97 | 0.87 |
| Income <133% of FPL | 1.90 | 0.15 | 1.29 | 0.45 | 1.01 | 0.98 | 0.96 | 0.86 | 1.29 | 0.28 | 0.81 | 0.54 | 0.91 | 0.71 | 1.62 | 0.03 | 1.49 | 0.24 |
| Income 133% -199% of FPL | 0.79 | 0.63 | 1.27 | 0.39 | 1.35 | 0.31 | 0.58 | 0.06 | 0.74 | 0.19 | 0.75 | 0.34 | 1.07 | 0.81 | 2.11 | 0.00 | 1.49 | 0.23 |
| Income 200-299% of FPL | 0.62 | 0.41 | 1.40 | 0.33 | 1.00 | 1.00 | 0.82 | 0.42 | 0.85 | 0.44 | 0.61 | 0.15 | 0.76 | 0.23 | 1.98 | 0.00 | 1.41 | 0.27 |
| Income 300-399% of FPL | 1.40 | 0.63 | 2.03 | 0.10 | 2.21 | 0.02 | 1.28 | 0.42 | 1.15 | 0.64 | 1.12 | 0.77 | 0.64 | 0.08 | 1.08 | 0.74 | 1.15 | 0.72 |
| >1 CSHCN | 1.20 | 0.54 | 0.77 | 0.29 | 0.72 | 0.19 | 0.9 | 0.52 | 1.20 | 0.20 | 0.78 | 0.27 | 1.15 | 0.42 | 1.13 | 0.33 | 0.90 | 0.56 |
| 1 other non-CSCHCN | 0.67 | 0.24 | 1.29 | 0.25 | 1.12 | 0.66 | 0.99 | 0.94 | 1.14 | 0.35 | 1.17 | 0.52 | 0.92 | 0.65 | 0.93 | 0.59 | 1.31 | 0.13 |
| 2 other non-CSCHCN | 0.56 | 0.16 | 0.96 | 0.90 | 1.01 | 0.96 | 1.12 | 0.59 | 1.14 | 0.45 | 0.67 | 0.15 | 1.14 | 0.49 | 1.13 | 0.41 | 0.93 | 0.74 |
| Single mother | 0.96 | 0.88 | 1.12 | 0.56 | 1.47 | 0.09 | 1.27 | 0.13 | 0.83 | 0.16 | 1.22 | 0.32 | 1.28 | 0.14 | 0.87 | 0.23 | 0.98 | 0.90 |
| Other family structure | 1.33 | 0.58 | 0.70 | 0.47 | 2.13 | 0.02 | 2.01 | 0.02 | 0.89 | 0.67 | 0.89 | 0.77 | 0.81 | 0.48 | 0.80 | 0.26 | 0.61 | 0.19 |
| Missing fam structure | 0.15 | 0.00 | 0.84 | 0.60 | 3.66 | 0.02 | 0.75 | 0.43 | 0.70 | 0.23 | 1.66 | 0.41 | 1.21 | 0.57 | 0.78 | 0.43 | 0.82 | 0.57 |
| < high school graduate | 0.71 | 0.32 | 0.92 | 0.67 | 1.00 | 0.99 | 1.14 | 0.44 | 1.02 | 0.89 | 0.93 | 0.75 | 0.77 | 0.11 | 0.95 | 0.71 | 0.85 | 0.35 |
| 5 or younger | 0.79 | 0.59 | 0.93 | 0.79 | 1.19 | 0.50 | 1.15 | 0.52 | 1.04 | 0.81 | 1.03 | 0.92 | 0.86 | 0.49 | 0.47 | 0.00 | 0.40 | 0.00 |
| 6 to 11 years old | 0.98 | 0.95 | 0.85 | 0.46 | 0.85 | 0.49 | 0.95 | 0.77 | 1.06 | 0.69 | 0.91 | 0.64 | 0.75 | 0.08 | 0.85 | 0.16 | 0.88 | 0.45 |
| Male child | 0.94 | 0.84 | 0.79 | 0.25 | 0.80 | 0.27 | 0.97 | 0.86 | 0.96 | 0.72 | 1.16 | 0.44 | 1.01 | 0.94 | 0.96 | 0.71 | 0.92 | 0.62 |
| Rural location | 0.37 | 0.02 | 1.11 | 0.63 | 0.72 | 0.20 | 2.18 | 0.00 | 0.78 | 0.14 | 0.79 | 0.37 | 1.09 | 0.64 | 0.99 | 0.95 | 1.06 | 0.78 |
| Missing location | 1.94 | 0.07 | 0.65 | 0.05 | 1.04 | 0.87 | 1.45 | 0.04 | 0.82 | 0.16 | 0.98 | 0.92 | 1.22 | 0.27 | 0.89 | 0.35 | 1.45 | 0.03 |
| Non-Hispanic Black | 1.11 | 0.79 | 0.86 | 0.57 | 0.55 | 0.08 | 1.51 | 0.07 | 0.84 | 0.33 | 0.94 | 0.83 | 1.67 | 0.02 | 0.62 | 0.01 | 0.49 | 0.01 |
| Hispanic | 1.38 | 0.42 | 0.86 | 0.56 | 0.98 | 0.95 | 0.93 | 0.76 | 0.92 | 0.68 | 1.60 | 0.12 | 1.67 | 0.10 | 0.60 | 0.01 | 0.86 | 0.58 |
| Other race/ethnicity | 0.52 | 0.19 | 1.21 | 0.53 | 1.14 | 0.70 | 1.19 | 0.44 | 0.94 | 0.79 | 1.30 | 0.46 | 1.34 | 0.21 | 1.31 | 0.14 | 1.10 | 0.73 |
| Primary language non-English | 0.55 | 0.30 | 0.78 | 0.53 | 1.41 | 0.43 | 0.57 | 0.14 | 1.31 | 0.34 | 1.30 | 0.54 | 1.84 | 0.06 | 1.49 | 0.14 | 0.94 | 0.87 |
| F statistic | 3.13 | | 2.82 | | 4.72 | | 4.54 | | 1.53 | | 2.00 | | 1.98 | | 7.97 | | 3.78 | |
